# Combination of Tolfenamic acid and curcumin induces colon cancer cell growth inhibition through modulating specific transcription factors and reactive oxygen species

**DOI:** 10.18632/oncotarget.6553

**Published:** 2015-12-10

**Authors:** Umesh T. Sankpal, Ganji Purnachandra Nagaraju, Sriharika R. Gottipolu, Myrna Hurtado, Christopher G. Jordan, Jerry W. Simecka, Mamoru Shoji, Bassel El-Rayes, Riyaz Basha

**Affiliations:** ^1^ Institute for Cancer Research, University of North Texas Health Science Center, Fort Worth, TX 76107, USA; ^2^ Texas College of Osteopathic Medicine, University of North Texas Health Science Center, Fort Worth, TX 76107, USA; ^3^ Department of Hematology Medical Oncology, Winship Cancer Institute, Emory University, Atlanta, GA 30322, USA; ^4^ JPS Center for Cancer Care, Fort Worth, TX 76104, USA; ^5^ Preclinical Services, University of North Texas Health Science Center, Fort Worth, TX 76107, USA; ^6^ College of Pharmacy, University of North Texas Health Science Center, Fort Worth, TX 76107, USA

**Keywords:** colon cancer, tolfenamic acid, curcumin, Sp1, NF-kB

## Abstract

Curcumin (Cur) has been extensively studied in several types of malignancies including colorectal cancer (CRC); however its clinical application is greatly affected by low bioavailability. Several strategies to improve the therapeutic response of Cur are being pursued, including its combination with small molecules and drugs. We investigated the therapeutic efficacy of Cur in combination with the small molecule tolfenamic acid (TA) in CRC cell lines. TA has been shown to inhibit the growth of human cancer cells *in vitro* and *in vivo*, via targeting the transcription factor specificity protein1 (Sp1) and suppressing survivin expression. CRC cell lines HCT116 and HT29 were treated with TA and/or Cur and cell viability was measured 24–72 hours post-treatment. While both agents caused a steady reduction in cell viability, following a clear dose/time-dependent response, the combination of TA+Cur showed higher growth inhibition when compared to either single agent. Effects on apoptosis were determined using flow cytometry (JC-1 staining to measure mitochondrial membrane potential), Western blot analysis (c-PARP expression) and caspase 3/7 activity. Reactive oxygen species (ROS) levels were measured by flow cytometry and the translocation of NF-kB into the nucleus was determined using immunofluorescence. Results showed that apoptotic markers and ROS activity were significantly upregulated following combination treatment, when compared to the individual agents. This was accompanied by decreased expression of Sp1, survivin and NF-kB translocation. The combination of TA+Cur was more effective in HCT116 cells than HT29 cells. These results demonstrate that TA may enhance the anti-proliferative efficacy of Cur in CRC cells.

## INTRODUCTION

Colorectal cancer (CRC) is the third leading causes of mortality among both males and females in the United States. It is estimated that 69,090 men and 63,610 women will be diagnosed with colorectal cancer in 2015 and 26,100 men and 23,600 women will die of the disease [1]. Despite the advances made in understanding the etiology of this disease and the incorporation of novel chemotherapy and biological agents, the prevalence of colon cancer and the mortality associated with this malignancy are very high and remain a serious concern in cancer care. Early detection and treatment can immensely affect the survival of patients (5-year survival rate of up to 90%). However, in patients with metastasis the survival rate drops to 10% [2]. Almost half of the patients with colon cancer will develop recurrent disease, suggesting that there is potential for improvement in current treatment regimens and the need to develop novel therapies. Standard treatment includes surgery followed by adjuvant therapy with oxaliplatin plus 5-fluorouracil, (FOLFOX) and leucovorin [3]. Although chemotherapeutic regimens containing FOLFOX produce a response in the majority of cases, virtually all responses are incomplete and the emergence of resistance, with subsequent recurrence, can occur in the majority of cases. Hence, there is a need to identify novel compounds that are non-toxic and that can be used in combination therapies to enhance the response of cancer cells to chemotherapeutic agents.

Recently there has been an increasing interest in using compounds of natural origin, particularly from dietary sources, for the treatment and prevention of various diseases including cancers [4]. Curcumin (Cur), in particular, has been extensively tested in preclinical studies for its therapeutic effect in several cancers including colon, pancreatic, prostate, gastric, hepatic, and leukemia [5]. Cur (diferuloylmethane) is the major active ingredient of turmeric derived from *Curcuma longa*, abundantly used in Southeast Asian countries. It has no discernable toxicity and has been shown to inhibit the growth of transformed cells and colon carcinogenesis at the initiation, promotion and progression stages in carcinogen-induced rodent models [6, 7]. Cur has also been shown to prevent the development of adenomas in the intestinal tract of Min +/− mice, a model of human familial adenomatous polyposis [8]. Cur is currently in multiple clinical trials. In a phase I and II clinical trials, curcumin has been found to be effective in inhibiting the growth of a variety of tumors [9–11], and shown to significantly reduce aberrant crypt foci formations that act as biomarkers for colon carcinogenesis [12]. Besides its anticancer activity, studies have demonstrated its chemopreventive properties *in vitro* and in animal models [13].

The anti-cancer and preventive properties of Cur are partly attributed to its ability to inhibit COX-2. Numerous studies have also demonstrated that COX-2 plays an important role in the development of colorectal cancer. A recent study demonstrated that the combination of the non-steroidal anti-inflammatory drug (NSAID) celecoxib (a specific COX-2 inhibitor) or its structural analog SC236 with Cur resulted in synergistic growth inhibition of colon cancer cells [14]. The possible mechanism involves both COX-2-dependent induction of apoptosis along with non-COX-2-dependent pathways. Similar results were also observed with the combination of another NSAID, diclofenac with Cur [15]. However, studies have also clearly demonstrated that the use of traditional NSAIDs or COX-2 inhibitors is associated with increased risk of gastrointestinal damage and adverse cardiovascular events. Therefore, this study was carried out to examine the effects of combining Cur and tolfenamic acid (TA) on colon cancer cells. TA is an NSAID that is primarily used in the treatment of migraine headaches [16, 17]. Research from our laboratory and others has demonstrated the anticancer activity of TA in various cancers including prostate, lung, ovarian, pancreatic, and pediatric cancers like neuroblastoma, medulloblastoma, and leukemia [18–24]. TA is currently being investigated in a Phase I clinical trial along with gemcitabine and radiation for treating pancreatic cancer patients. The rationale for using TA in this study includes its limited side effects and an overlap in pathways that are also targeted by Cur, namely then NF-κB signaling and the Sp1 transcription factor [25–27].

In this study we found that the combination of Cur and TA resulted in an increased inhibition of CRC cell growth via the induction of apoptosis when compared to individual agents. This investigation also revealed the efficacy of the combination treatment in modulating the expression of transcription factors Sp1 and NF-κB and anti-apoptotic protein survivin, altering ROS levels and mitochondrial membrane potential and inhibiting the nuclear translocation of NF-κB.

## RESULTS

### Combination of Cur and TA results in increased inhibition of cell growth

**Figure 1:**
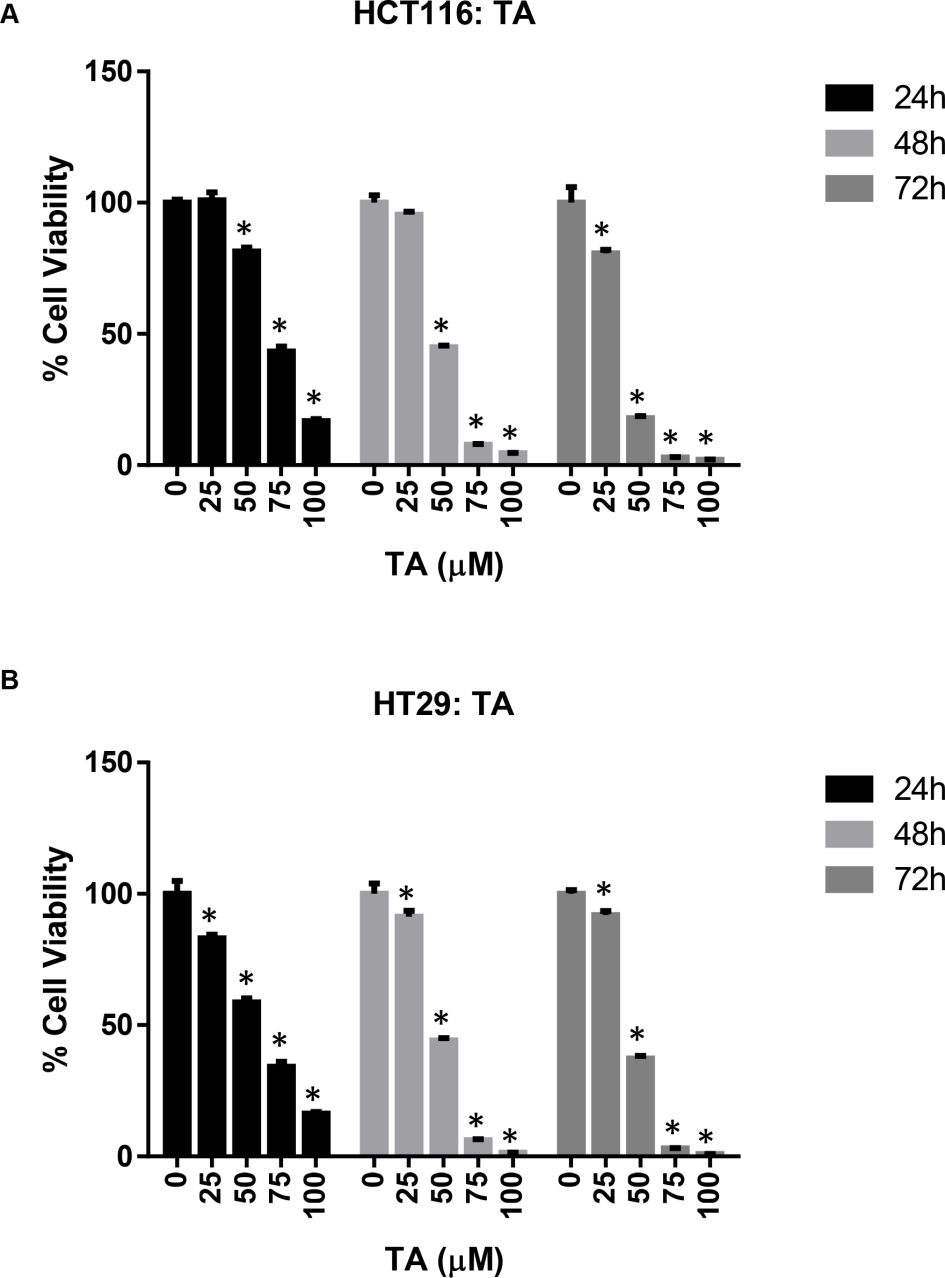
Anti-proliferative activity of TA in CRC cell lines HCT116 (**A**) and HT29 (**B**) cells were treated with DMSO (Control) or increasing concentrations of TA (25-100 μM) for 24-72 h. Cell viability was determined using CellTiter-Glo cell viability assay and results are presented as % viable cells (relative to control). Values represent mean ± SEM (*n* = 3) and the bars with ‘*’ are significantly different from control (*p* < 0.05).

**Figure 2:**
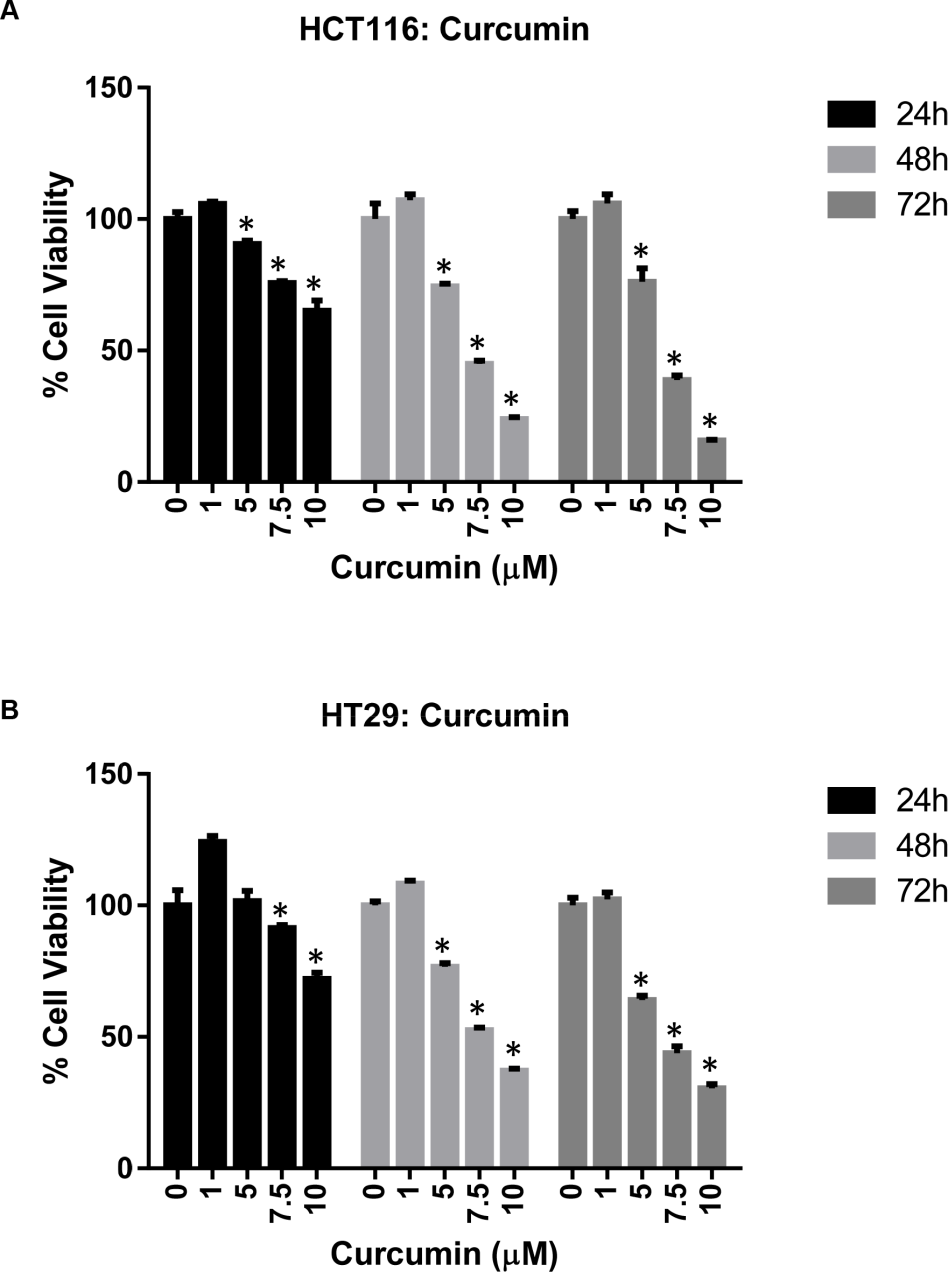
Anti-proliferative activity of Cur in CRC cell lines HCT116 (**A**) and HT29 (**B**) cells were treated with DMSO (Control) or Cur (1-10 μM). Cell viability was measured at 24-72 h post-treatment. Results are presented as % viable cells compared to control. Each value represents mean ± SEM of 3 independent determinations. Bars marked with ‘*’ are significantly different from control (*p* < 0.05).

The effect of TA and Cur on CRC (HCT116 and HT29) cell proliferation was evaluated by the CellTiter- Glo luminescent cell viability assay. Data from the dose graphs was used to calculate the IC_50_ values (Supplementary Figures S1 & S2) for the individual drugs. Based on these results, the doses for TA and Cur were selected to test the combination effect. HCT116 and HT29 cells were treated with increasing concentrations of TA (0, 25, 50, 75, 100 μM) or Cur (0, 1, 5, 7.5, 10 μM). Cell viability was evaluated at 24, 48, and 72 h post-treatment using the CellTiter-Glo assay kit as described in the methods (Figures 1 and 2). In HCT116 cells, the IC_50_ values for TA and Cur were 70.3 μM and 13.46 μM at 24 h and 47.8 μM and 3.6 μM at 48 h, respectively. In HT29 cells, the IC_50_ values for TA and Cur were 55.3 μM and 12.9 μM at 24 h and 46.8 μM and 4.7 μM at 48 h, respectively. As seen in Figures 1 and 2, TA and Cur decreased cell viability in a dose and time-dependent manner that demonstrates their potent anti-cancer activity IC_50_.

**Figure 3:**
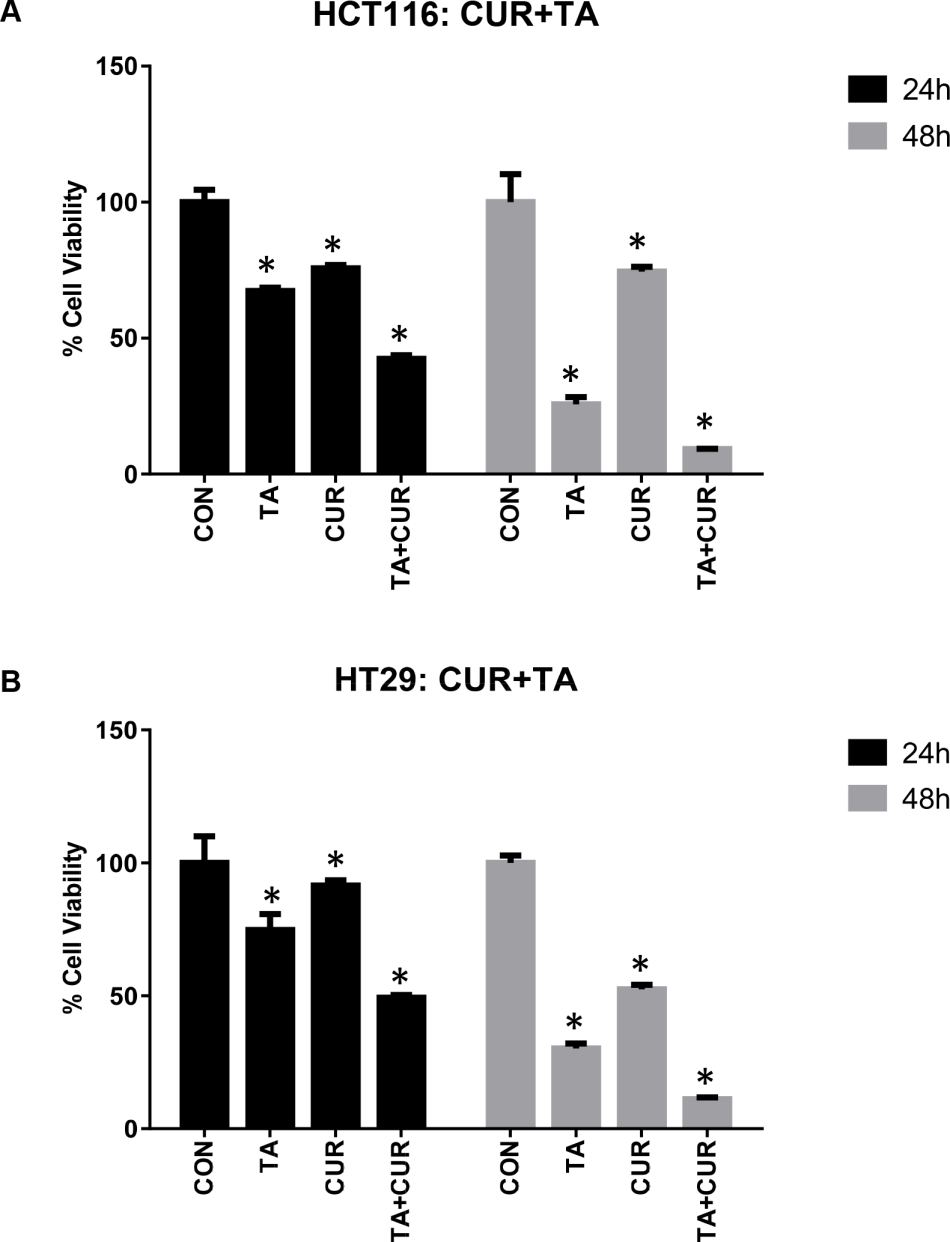
Anti-proliferative activity of TA+Cur in CRC cell lines CRC cells, HCT116 (**A**) and HT29 (**B**) were treated with DMSO (Control) or TA (50 μM) or Cur (7.5 μM) or TA+Cur. Cell viability was determined at 24 and 48 h post-treatment. Data shown indicates % viable cells relative to control. Bars indicate mean ± SEM (*n* = 3) and the bars marked with ‘*’ are significantly different from the corresponding control (*p* < 0.05).

To examine the effects of combined treatment with TA+Cur, HCT116 and HT29 cells were treated with 50 μM TA and 7.5 μM Cur and viable cells were measured at 24 and 48 h post-treatment. Interestingly, combination treatment resulted in a reduction of cell viability in both HCT116 (24 h: 58%; 48 h: 91%) and HT29 (24 h: 51%; 48 h: 89%) cells (Figure 3). These results suggest that the combination treatment was more effective in inhibiting the proliferation of both HCT116 and HT29 cells compared to single treatment with TA or Cur.

**Figure 4:**
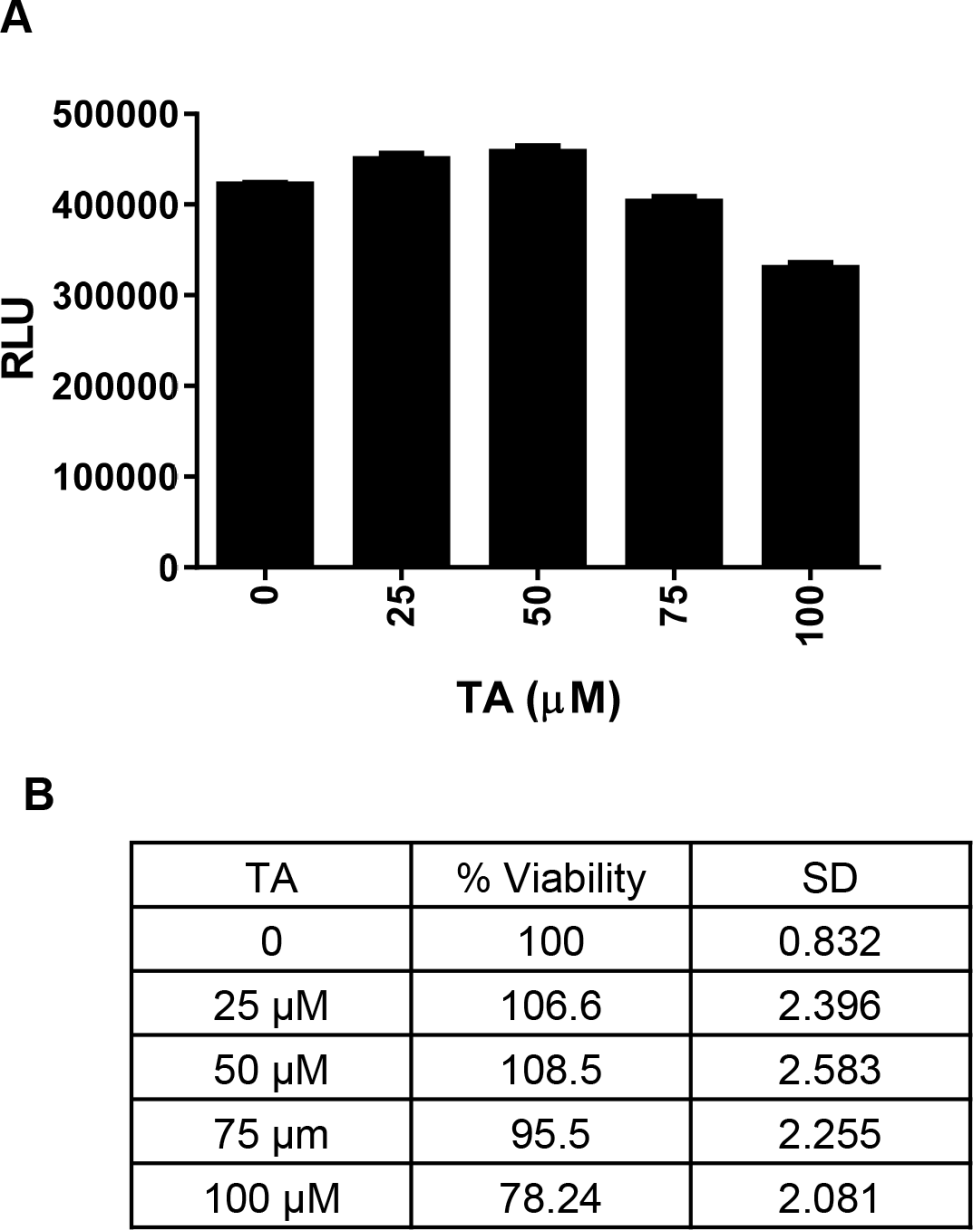
Effect of TA on cardiomyocytes proliferation H9C2 cardiomyocyte cells were treated with DMSO (Control) or increasing concentrations of TA (25-100 μM) and cell viability was determined at 48 h post-treatment. Data shown indicates (**A**) Mean ± SEM (*n* = 3) of actual luminescence values (RLU-Relative Light Units) and (**B**) Percent viable cells (relative to control).

To address issues of NSAID associated cardio-toxicity, we treated cardiomyocytes H9C2 with increasing dose TA (0, 25, 50, 75, 100 μM) for 48 h. As seen in Figure 4, TA did not have any cytotoxic effect on these cells.

### Combination treatment results in loss of mitochondrial membrane potential and induces PARP cleavage and survivin downregulation

**Figure 5:**
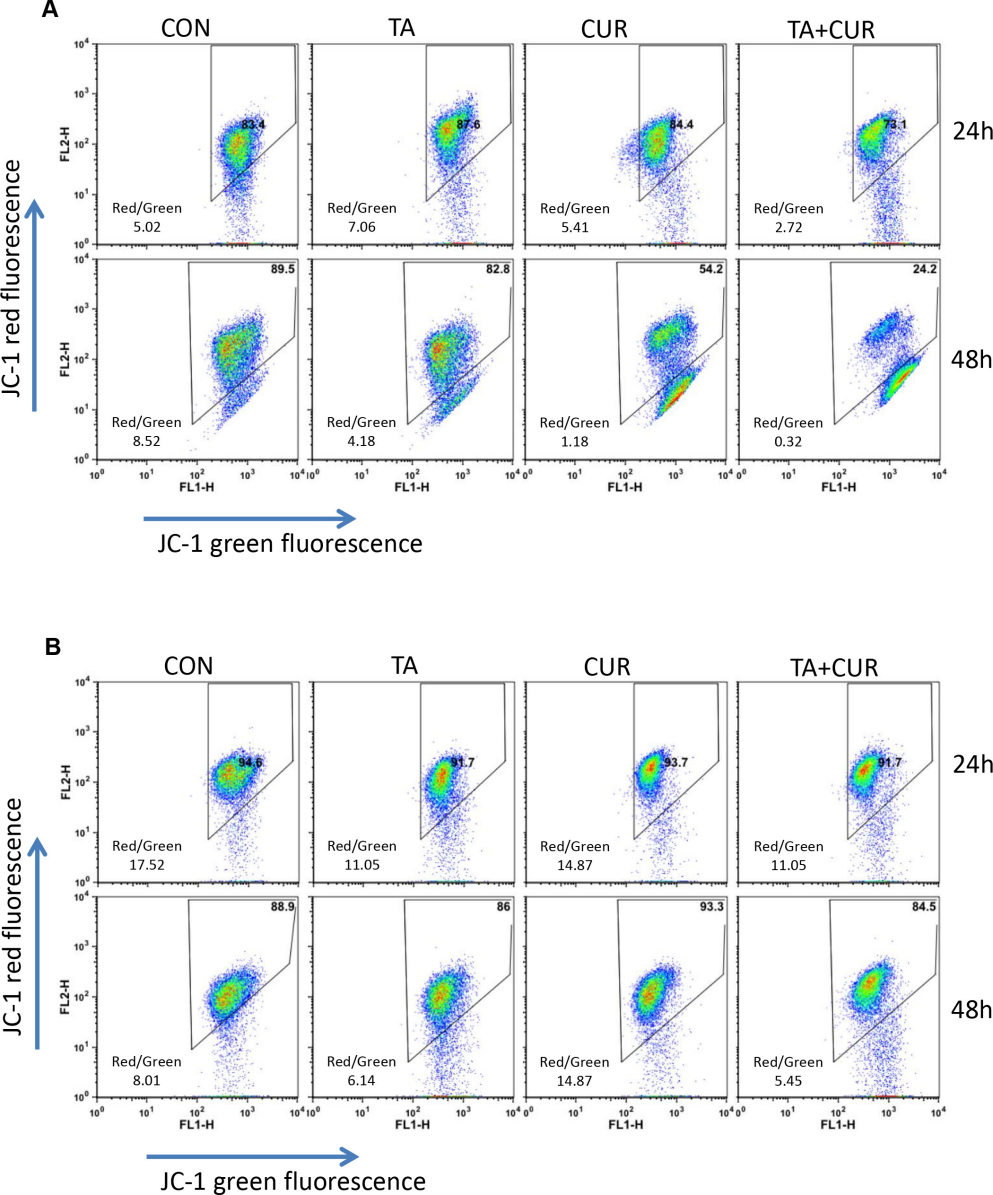
TA and Cur treatment results in loss of membrane potential HCT116 (**A**) and HT29 (**B**) cells were treated with DMSO (control) or TA (50 μM) or Cur (7.5 μM) or TA+Cur for 24 and 48 h. The loss of membrane potential was measured using the JC1 membrane potential kit (Invitrogen) on BD FACSCalibur flow cytometer and data were analyzed using FlowJo software.

Mitochondria play a key role in propagating apoptotic signals. Induction of apoptosis via mitochondrial pathways results in the loss of mitochondrial membrane potential. We therefore examined the effect of TA and/or curcumin on the change in mitochondrial potential (Δψm) by JC-1 staining. HCT116 and HT29 cells were treated with TA or Cur alone or in combination and the changes in membrane potential were assessed at 24 and 48 h post-treatment using the JC-1 dye. Quantitative analysis using flow cytometry revealed that the percentage of cells with loss of Δψm increased following treatment with TA+Cur, (Figure 5). The disruption of membrane potential (ratio of red and green fluorescence) in response to TA+Cur treatment showed a clear trend in HCT116 cells (Con: 5.02 vs TA+Cur: 2.72); however this trend was not obvious in HT29 cells (Con: 17. 52 vs TA+Cur: 11.05).

### Cur and TA combination induces caspase 3/7 activity

**Figure 6:**
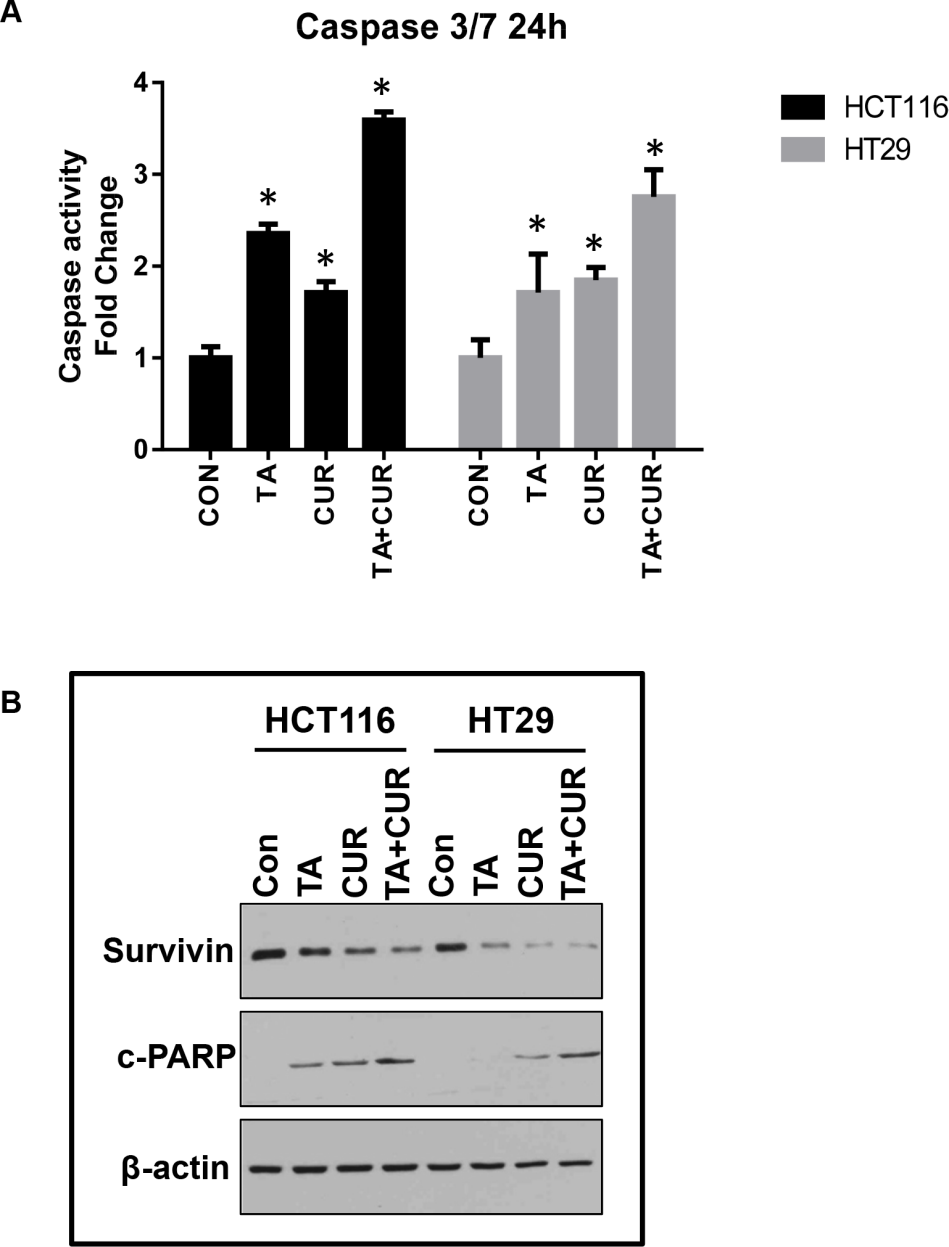
Cur and TA combination induces apoptotic markers CRC cell lines, HCT116 and HT29 were treated with DMSO (Control) or TA (50 μM) or Cur (7.5 μM) or combination of TA+Cur for 24 h. (**A**) The activity of caspase 3 and 7 was measured 24 h post-treatment using Caspase 3/7-Glo kit. Bars represent mean ± SEM (*n* = 3) and the bars marked with ‘*’ are significantly different from the corresponding control (*p* < 0.05). (**B**) Cells were treated for 24 h as described above and protein extracts were prepared. The expression of survivin and c-PARP was determined by Western blot analysis. β-actin was used as a loading control.

To determine whether the decrease in cell proliferation with the combination treatment was associated with an induction of apoptotic markers, activity of caspase 3 and 7 was measured. HCT116 and HT29 cells were treated with TA (50 μM) and Cur (7.5 μM) alone and in combination and caspase 3/7 activity was measured using the Caspase-Glo 3/7 assay kit, at 24 and 48 h post-treatment. As shown in Figure 6A, caspase 3/7 activity was markedly increased in cells treated with the combination of TA+Cur (3.6 and 2.7 fold) compared to single agent treatment with TA (2.3 and 1.7 fold) or Cur (1.7 and 1.8 fold) in HCT116 and HT29 cells, respectively. These results corroborated the findings of cell viability (Figure 3), suggesting the association of decreased proliferation of cells with the induction of apoptosis.

We also looked at the downstream targets of the apoptotic activation cascade, such as cleavage of PARP protein, and levels of the anti-apoptotic protein survivin, using Western blot (Figure 6B). We observed an increase in the levels of cleaved PARP in both HCT116 and HT29 cells at 24 h post-treatment. Also, levels of survivin were decreased in cells treated with TA+Cur combination compared to single drug treatments. Taken together, these results confirm that the inhibition of proliferation by the combination treatment could be the result of activation of apoptosis as demonstrated by an increase in caspase 3/7 activity and PARP cleavage and the downregulation of the anti-apoptotic protein survivin.

### Induction of ROS levels following cur treatment

**Figure 7:**
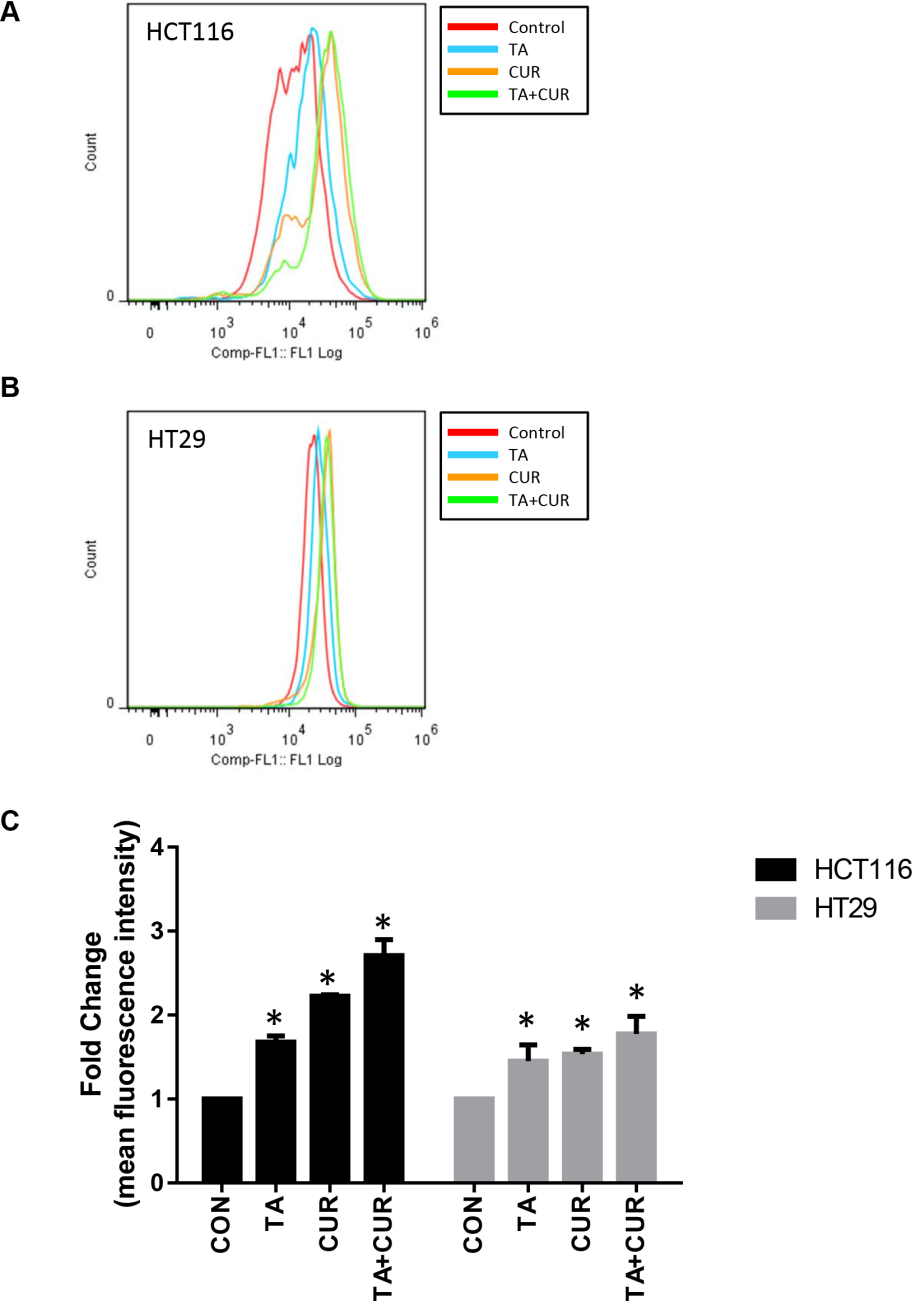
Effect of TA and Cur treatment on the generation of ROS in CRC cells (**A**) HCT116 and (**B**) HT29 cells were treated with TA (50 μM) or Cur (7.5 μM) or combination of TA+Cur for 24 h and ROS levels were measured with CM-H2DCFDA (Invitrogen) kit using flow cytometry. Data were analyzed using FlowJo software. (**C**) Fold change in mean fluorescence intensity (MFI) was used to plot the graphs. Bars (mean ± SEM) represent fold-change over control and bars marked with ‘*’ are significantly different (*p* < 0.05) from corresponding controls.

The effect of Cur, TA or TA+Cur on ROS levels was determined in both CRC cell lines following 24 h treatment. While both TA and Cur increased the levels of ROS in both cell lines, the combination of TA+Cur led to a much greater elevation than either individual agent (Figure 7). TA increased ROS by 1.6 and 1.4 fold, respectively, in HCT116 and HT29 cells. Cur caused 2.2 (HCT116) and 1.5 (HT29) fold increase while the combination treatment induced ROS levels by 2.7 fold in HCT116 and 1.8 fold in HT29 cells.

### Cur and TA treatment downregulates Sp1 and NF-κB transcription factors

**Figure 8:**
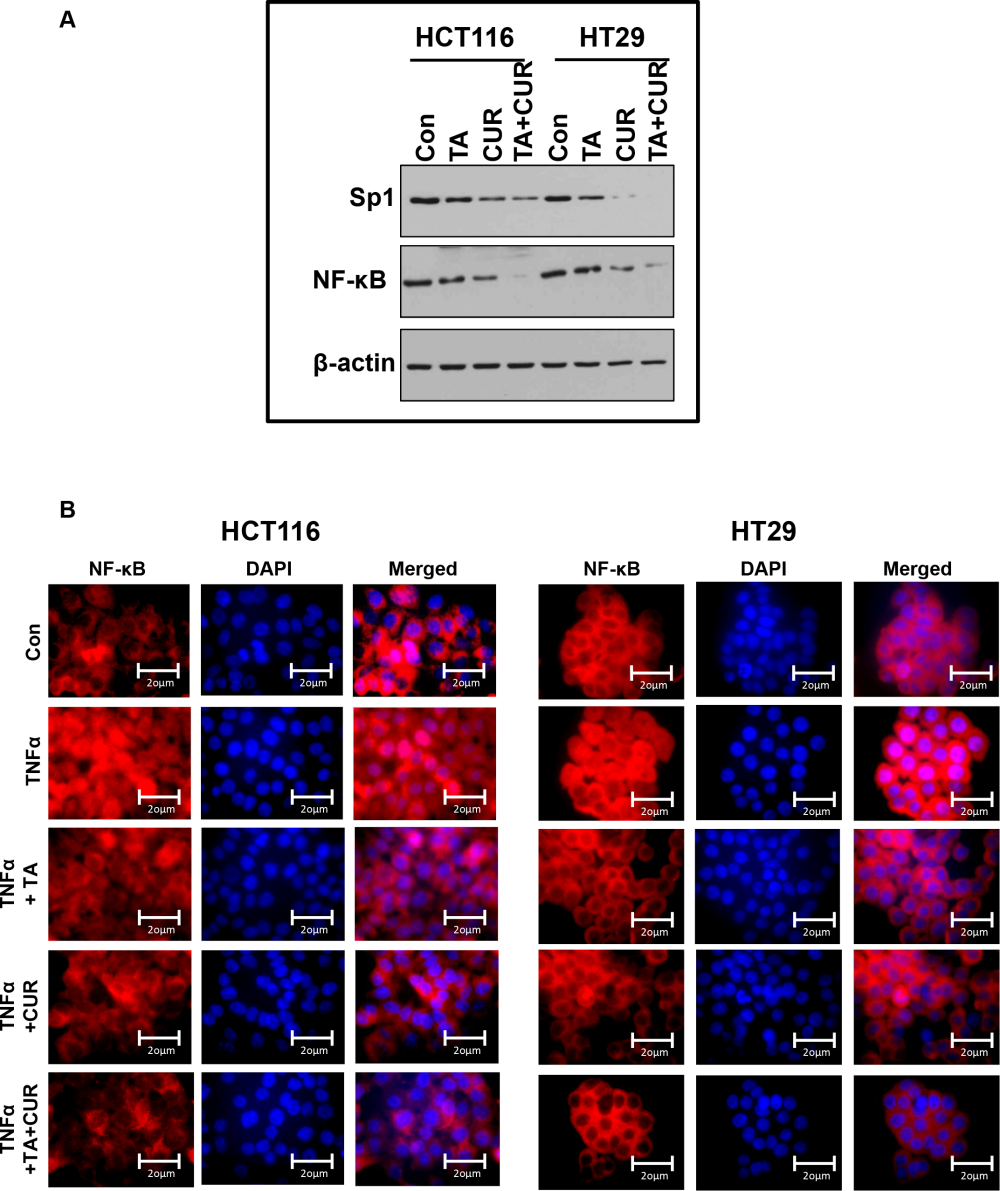
NF-kB translocation in CRC cells following TA and Cur treatment (**A**) HCT116 and HT29 cells were treated with TA (50 μM) or Cur (7.5 μM) or TA+Cur for 24 h. Protein extracts were prepared and the expression of Sp1 and NF-kB was determined by Western blot analysis. β-Actin was used as a loading control. (**B**) HCT116 and HT29 cells were treated with DMSO (Con) or TA or Cur or combination of TA+Cur in the presence of TNFα (15 ng/ml). Cells were processed to examine the translocation of NF-kB into the nucleus by immunofluorescence. Images were captured at 60X magnification using Olympus AX70 upright fluorescence microscope and representative sections are presented in the figure. Con: Control; TA: Treated with Tolfenamic acid; Cur: Curcumin; TA+CUR: Tolfenamic acid and curcumin.

To further examine the mechanism by which the combination of TA+Cur inhibits cell proliferation of HCT116 and HT29 cells, we examined their effect on some of the known key proteins/pathways targeted by the individual agents. Previous studies with TA or Cur have shown that they both target the Sp transcription factor and the NF- κB signaling pathway in colon cancer cells. The effect of combination treatment on Sp1 and NF-κB expression was determined by Western blot. As shown in Figure 8A, TA and Cur treatment alone decreased the expression of Sp1 in both cell lines and this effect was augmented following the combination treatment. NF- κB expression was downregulated only in Cur treated cells, but not in TA treated cells. However, a significant reduction in NF-κB levels was seen in cells treated with a combination of both TA+Cur.

The effect of these treatments on TNFα induced translocation of NF-kB to the nucleus was evaluated by immunofluorescence. The examination of NF-kB staining in the cytoplasm and nucleus of cells revealed certain differences between treated samples. As seen in Figure 8B, TNFα treatment results in the translocation of NF-κB (red fluorescence) from the cytoplasm into the nucleus (blue, DAPI stained). Both TA and Cur are able to inhibit this translocation. However, the combination of TA+Cur resulted in a significantly decreased translocation of NF-κB from the cytoplasm to nucleus when compared to control, TA, or Cur alone treated cells.

## DISCUSSION

Cur has been extensively characterized for its anti-cancer activity in various cancers including CRC [5, 28]. Cur is an attractive therapeutic molecule as it displays a broad spectrum of activities and affects multiple proteins and pathways during the pre-initiation, initiation, and progressive stages of colon cancer. One of the major problems associated with the clinical use of Cur is its poor systemic availability [29]. To improve the bioavailability of Cur various strategies are being explored including novel formulations for delivery [30], synthesizing structural analogs with more potent activity and increased bioavailability [31]. Studies are also being carried out to increase the therapeutic efficacy by using combination therapy of Cur with chemotherapeutic drugs [32–34]. In this study we used a combination of TA and Cur. Both TA and Cur showed the ability to inhibit the growth of CRC cells and this inhibition is significantly increased when both agents are combined together (Figures 1–3). The loss of cell viability also correlated with loss of membrane potential (Figure 5) and an increase in apoptosis (Figure 6). We also carried out studies to determine if the effect is synergistic or additive or antagonistic with the combination of TA and Cur, by calculating the combination index (CI) using two different combinations. At the IC_50_ doses for TA and Cur we observed an additive response (CI – 0.9–1.1) between the two drugs in both the cells lines. At the doses (TA: 50 μM; Cur: 7.5 μM) used for the experiments in this manuscript, we observed additive response only in HCT116 cells. In HT29 cells, CI was not showing additive or synergistic effect; however the combination of TA+Cur resulted in higher growth inhibitory response when compared to single agent.

NF-κB is a ubiquitous transcription factor that is present in the cytoplasm. In response to various inflammatory stimuli, NF-κB translocates into the nucleus where it is known to transcriptionally activate several genes involved in inflammation, cell proliferation, invasion, metastasis, angiogenesis, and suppression of apoptosis [35]. Its association with cancer cell survival and resistance to chemotherapy makes it an attractive target for anti-cancer therapies. In CRC, both TA and Cur have been shown to modulate the NF-κB signaling pathway [25, 26, 36]. In the presence of inflammatory stimuli, TA prevented inflammatory cytokine-induced NF- κB activation by inhibiting its translocation to the nucleus [25]. It has also been reported that TA has the ability to downregulate NF-κB expression in HCT116 colon cancer cells [37]. In this study, we found that TA alone did not affect the levels of NF-κB; however the combination of TA+Cur inhibited NF-kB protein expression more than that caused by Cur alone (Figure 8A), suggesting that TA enhances the ability of Cur to downregulate NF-κB. We also observed that inflammatory stimuli-induced (TNFα) nuclear translocation of NF-κB was significantly inhibited by both TA and Cur and this effect was further enhanced by the combination treatment of TA+Cur (Figure 8B).

Survivin is a member of the inhibitor of apoptosis protein (IAP) family and is highly expressed in various human cancers, including colon cancer, and its expression correlates with tumor stage and poor progression [38, 39]. Survivin has also been shown to regulate mitotic spindle checkpoint, angiogenesis, and cause resistance of tumor cells to radiation and chemotherapy, making it an ideal tumor marker and a therapeutic target [40]. NSAIDs with anti-cancer activity such as celecoxib and indomethacin have been shown to inhibit the expression of survivin [41, 42]. Research from our laboratory has shown that TA (small molecule and NSAID) downregulates the expression of survivin in various cancers including pancreatic, prostate, ovarian, leukemia, and medulloblastoma [18, 20, 22, 24]. Promoter analysis of the survivin gene shows the presence of Sp1 binding sites, suggesting its regulation by the Sp1 transcription factor [43, 44]. We have shown that TA inhibits the expression of Sp1 transcription factor, which in turn might be responsible for regulating survivin expression. Studies have shown that Cur also has the ability to downregulate the expression of Sp transcription factors in CRC cells [36, 45]. In agreement with these reports, results from this study show that both Cur and TA downregulate survivin and Sp1 transcription factor in HCT116 and HT29 cells (Figures 6B, 8A). Results also demonstrate that combination treatment with TA+Cur caused greater downregulation of Sp1 compared to single drug treatment, resulting in an increase in the suppression of survivin. This downregulation of survivin by the combination treatment might contribute towards the apoptotic cell death.

Increase in ROS levels is known to play a role in tumor initiation and also acts as a chemotherapeutic strategy to inhibit tumor growth. Both Cur and TA have been shown to increase ROS levels in colon cancer cells. TA-induced ROS results in DNA damage and subsequent activation of NF-κB and ATF3 [46]. Curcumin on the other hand was shown to mediate the effect of increased ROS by downregulating microRNAs (miR-27a and miR-20a) which in turn results in decrease in Sp1 and its downstream targets [45]. Our results demonstrate that the combination of TA+Cur results in an increase in ROS levels compared to single drug treatment (Figure 7). We have also shown that Sp1 levels are downregulated by treatment with TA and Cur (Figure 8A). Taken together our results suggest that the increase in ROS by the combination treatment leads to inhibition of Sp1 expression. This is in agreement with the study showing that downregulation of Sp1 by Cur is ROS-dependent [45].

Both Cur and TA have shown anti-cancer activity in multiple cancers including CRC [18–20, 25, 47–49] by targeting NF-κB and Sp1 respectively. Interestingly, there is overlap in some of the targets for these two agents [25, 36, 45]. In this study, we demonstrated that the combination of Cur and TA enhanced the inhibition of Sp1, NF-kB and survivin and upregulated the levels of ROS and apoptotic markers such as PARP cleavage and effector caspase activity. We also show that TA, unlike other NSAIDs, did not exhibit any cardio-toxicity (Figure 4). These studies provide highly encouraging evidence to support testing of this novel combination to increase therapeutic efficacy in CRC. Cur is currently under investigation in 117 clinical trials for various human diseases and about half (52) of these studies (mostly in combination studies) are in cancer of different types (*clinicaltrials.gov*). TA is being explored in a Phase I clinical trial to test its activity in pancreatic cancer patients along with gemcitabine and radiation. Favorable results from studies of the TA and Cur combination in animal models will help move our experimental strategy into clinical practice.

## MATERIALS AND METHODS

### Cell culture and reagents

Human colorectal cancer cell lines, HCT116 and HT29 and cardiomyocytes H9C2 were purchased from the American Type Culture Collection (ATCC, Manassas, VA). Colon cancer cells were grown in McCoy's 5A medium, H9C2 cells were grown in DMEM medium and supplemented with fetal bovine serum and maintained in a humidified incubator at 37°C in an atmosphere of 5% CO_2_.

Tolfenamic acid, curcumin, and DMSO were purchased from Sigma (St. Louis, MO). Antibodies against cPARP and NF-κB were obtained from Cell Signaling Technologies (Beverly, MA), Sp1 and HRP-conjugated mouse anti-rabbit antibody from Santa Cruz (Santa Cruz, CA), survivin from R&D (Minneapolis, MN), and β-actin from Sigma.

### Cell proliferation assay

The effect of TA and Cur on cell proliferation was determined by measuring cellular ATP levels as an indication of viable cells using the CellTiter-Glo (luminescent cell viability assay kit from Promega, Madison, WI). Briefly, HCT116, HT29, and H9C2 cells were seeded in white-walled clear-bottom 96-well plates. After 24 h, cells were treated with solvent (DMSO) or various concentrations of TA and Cur alone and in combination. Cell viability was assessed at various time points by adding the CellTiter-Glo reagent (Promega) according to the manufacturer's instructions. Cells were treated in triplicate and data were normalized to solvent control and reported as percentage of viable cells.

### Caspase activation

The induction of apoptosis was determined by measuring caspase 3/7 activity using the Caspase-Glo 3/7 Assay kit (Promega). HCT116 and HT29 cells were seeded in 96-well plates and treated as described above. Activation of caspase 3 and 7 was measured by adding the Caspase-Glo 3/7 reagent following the manufacturer's instructions. Luminescence values from each well, corresponding to caspase 3/7 activity, were measured. Cells were treated in triplicate and data normalized to solvent control and reported as fold change in caspase 3/7 activity.

### Mitochondrial membrane potential

Depolarization of mitochondrial membrane potential and the subsequent activation of caspases is the hallmark of apoptosis. Mitochondrial membrane potential (Δψ) in cells treated with TA, Cur or TA+Cur combination was measured using the JC-1 dye (Invitrogen, Carlsbad, CA). The dye exists as a monomer at low concentrations with green fluorescence (E_m_ 530 nm) and accumulates in the mitochondria as aggregates with orange-red fluorescence (E_m_ 590 nm). During apoptosis there is a disruption in the mitochondrial membrane potential that can be monitored by the shift in fluorescence from red to green. Briefly, following treatment with TA, Cur or TA+Cur, cells were harvested and incubated with 5 μM JC1 dye for 15 min at 37°C. Cells were washed, resuspended in fresh medium and analyzed using a FACS Caliber flow cytometer (BD Biosciences, San Jose, CA). Data was analyzed using FlowJo software (Tree Star Inc, Ashland, OR) and represented as a dot plot of green versus red fluorescence. Apoptosis was determined by the increase in red fluorescence (aggregates) accompanied by loss of green fluorescence (monomers).

### Protein extraction and western blotting

Protein lysates for Western blot experiments were prepared using lysis buffer consisting of cell extraction buffer (Invitrogen) supplemented with protease inhibitor cocktail (Sigma). Briefly, cells were harvested at 24 and 48 h post-treatment and the cell pellets were re-suspended in cold cell lysis buffer, incubated for 30 min on ice, and centrifuged for 15 min at 12,000 rpm. Protein concentration in the supernatant (cell lysate) was estimated using BCA protein assay kit (Thermo Fisher Scientific, Waltham, MA). For Western blot, 25–30 μg protein was separated on 8 or 10% polyacrylamide gel, and transferred to a nitrocellulose membrane using iBlot transfer system (Invitrogen). The membrane was blocked with 5% milk-TBST and probed with primary antibodies against, Sp1, survivin, cPARP, NF-κB, and β-actin. Membranes were washed and incubated with horseradish peroxidase (HRP)-conjugated secondary antibody. The blots were developed using Supersignal West Dura Extended Duration Substrate (Thermo Fisher Scientific) and images acquired using BioSpectrum Imaging System (UVP).

### Estimation of reactive oxygen species

The levels of ROS were measured with an oxidative stress indicator, CM-H2DCFDA (Invitrogen) via flow cytometry. Briefly, CRC cells were treated with TA, Cur or TA+Cur for 24 h. H_2_O_2_ was used as a positive control to induce ROS. Cells were harvested and incubated with 5 μM of CM-H2DCFDA for 30 min at 37°C in the dark. Fluorescence was quantified by a BD FACSCalibur and the results were analyzed using FlowJo (Tree Star Inc, Ashland, OR). Geometric mean fluorescence was used as the average fluorescence value for each population.

### NF-kB immunofluorescence

HCT116 and HT29 colon cancer cells were grown on coverslips in a 6-well dish overnight. NF-κB translocation was induced by treating cells with TNFα (15 ng/ml). Cells were simultaneously treated with TA (50 μM), Cur (7.5 μM) or a combination of TA+Cur for 24 h. After treatment, cells were fixed with cold methanol for 10 min. Cells were washed with PBS and blocked with 10% normal goat serum for 1 hr. Cells were then incubated with NF-κB antibody (1:200) for 2 h, washed with 0.1% PBS-Tween and incubated with goat anti-rabbit antibody (1:400) conjugated with Alexa Fluor 594 for 2 h. Cells were washed with PBS and mounted on glass slides using Vectashield Mounting Medium with DAPI (Vector Laboratories, Burlingame, CA). Images were acquired using Olympus AX70 upright fluorescence microscope.

### Statistical analysis

All data were analyzed using one way ANOVA. Results were expressed as mean ± SEM. Significance between treated and untreated cells were evaluated and *p* value < 0.05 was considered significant.

## SUPPLEMENTARY MATERIAL FIGURES AND TABLES


