# Supplementary material for: Combination of Tolfenamic acid and curcumin induces colon cancer cell growth inhibition through modulating specific transcription factors and reactive oxygen species
Source: Oncotarget. 2015 Dec 10;7(3):3186–200. doi: 10.18632/oncotarget.6553 (PMC4823099; doi:10.18632/oncotarget.6553)
Supplement: Supplementary file 1 [file oncotarget-07-3186-s001.pdf]

Combination of Tolfenamic acid and curcumin induces colon cancer cell growth inhibition through modulating specific transcription factors and reactive oxygen species

Supplementary Materials

A

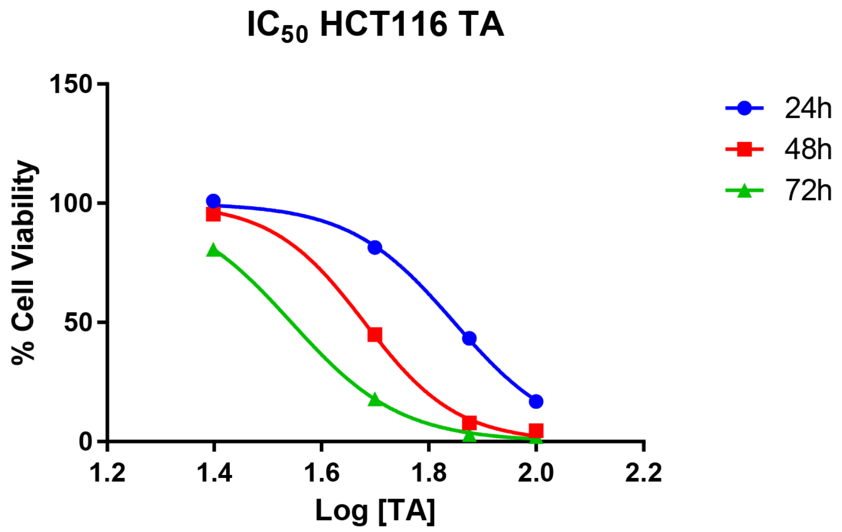

	24h	48h	72h
IC_{50} (µM)	70.31	47.83	34.96

B

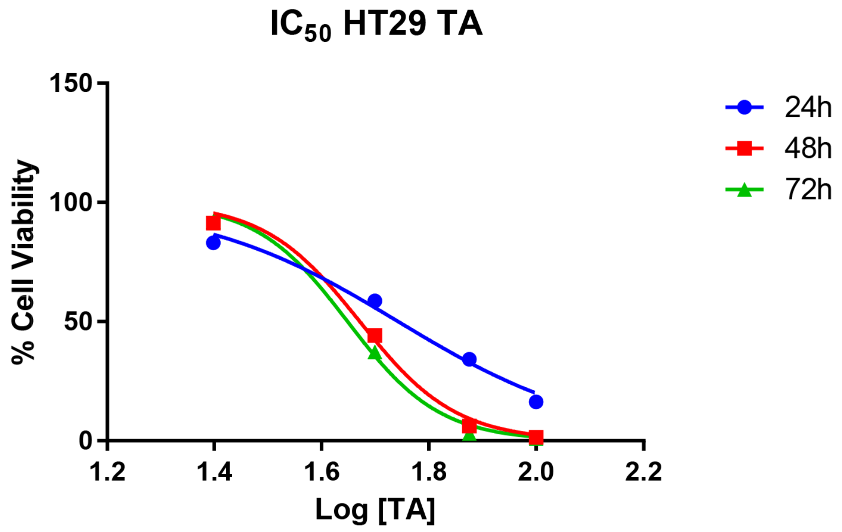

	24h	48h	72h
IC_{50} (µM)	55.29	46.89	44.50

A

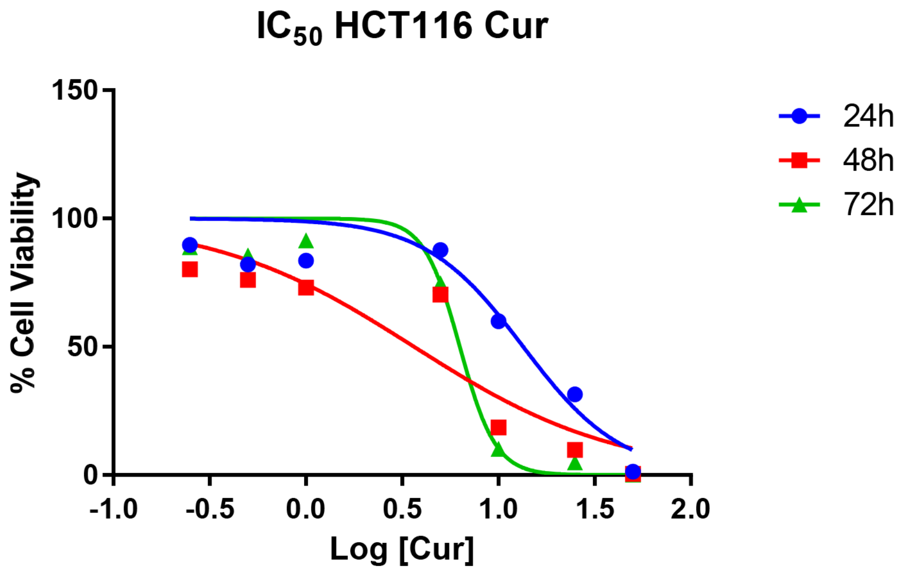

	24h	48h	72h
IC_{50} (µM)	13.46	3.648	6.303

B

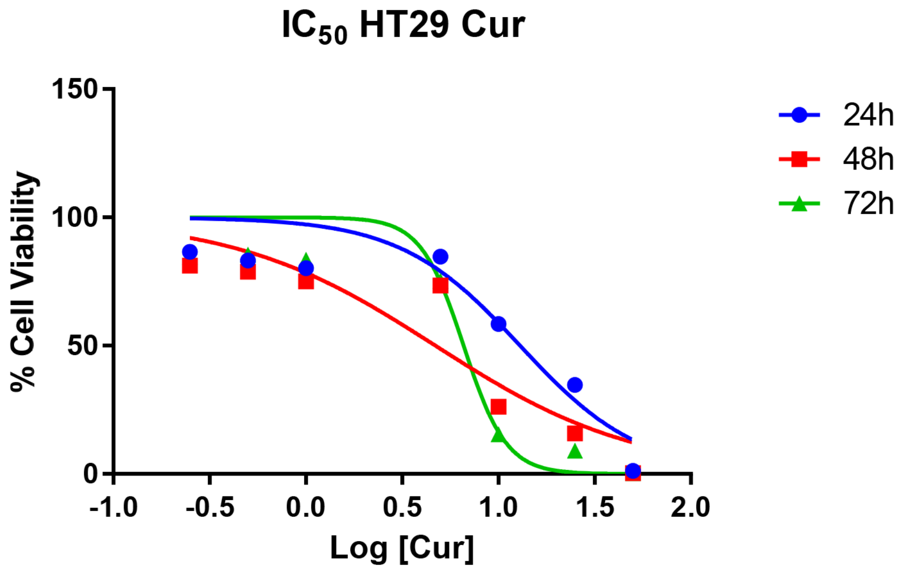

	24h	48h	72h
IC_{50} (µM)	12.95	4.706	6.596

Supplementary Figures S1 and S2: Determination of IC_{50} values for TA and Cur. HCT116 (**A**) and HT29 (**B**) cells were treated with DMSO (Control) or increasing concentrations of TA (25-100 µM) or Cur (1-10 µM) for 24-72 h. Cell viability was determined using CellTiter-Glo cell viability assay (Figures 1 and 2). Data from Figures 1 and 2 was used to calculate the IC_{50} values for TA and Cur at each time point by plotting the log (TA/CUR) vs normalized response curve using GraphPad Prism.
